# The effect of platelet–albumin ratio on mortality and morbidity in peptic ulcer perforation

**DOI:** 10.1097/MD.0000000000029582

**Published:** 2022-08-05

**Authors:** Hüseyin Bilge, Ömer Başol

**Affiliations:** a Department of General Surgery, Dicle University Faculty of Medicine, Diyarbakir/Turkey.

**Keywords:** hospital mortality, peptic ulcer perforation, serum albumin

## Abstract

**Background:**

The aim of our study was to investigate the prognostic role of platelet/albumin ratio in patients treated under emergency conditions for peptic ulcer perforation (PUP).

**Methods:**

A retrospective study involving emergency patients who were operated for PUP was carried out. The patients were divided into 2 groups: PUP patients who died after surgical treatment (PUP-M) and PUP patients who survived after surgical treatment (PUP-S). The laboratory values of the patients were compared statistically. A *P* value of <.05 was considered statistically significant.

**Results:**

This cohort study consisted of 171 patients treated between June 2013 and December 2019. The mean age of the patients was 46.3 ± 20.5 years; and 33 (19.3%) patients were women. The age (*P* ≤ .001), platelet/lymphocyte ratio (*P* = .02), lactic dehydrogenase to albumin ratio (*P* ≤ .001), and platelet/albumin ratio (PAR; *P* ≤ .001) values were high and lymphocyte count was low (*P* = .006) in the PUP-M group. A positive correlation was determined between length of stay in hospital and age (*P* ≤ .001), lactic dehydrogenase/albumin ratio (*P* ≤ .001), platelet count (*P* = .044), and PAR (*P* ≤ .001). A substantial negative correlation was determined between length of stay in hospital and albumin count (*P* ≤ .001).

**Conclusions:**

We determined a high preoperative PAR level in PUP patients who had undergone surgery as a negative prognostic parameter. PAR is a candidate biomarker for clinical practice.

## 1. Introduction

Peptic ulcer disease (PUD) is the most common gastrointestinal disease worldwide. Approximately 4 million people are affected each year.^[1]^ The global prevalence of PUD has decreased in recent years,^[2,3]^ but the complications from peptic ulcers have not decreased.^[3,4]^ The reduction in peptic ulcer disease is partly explained by helicobacter pylori eradication therapy. However, the complications of peptic ulcer disease, especially bleeding and perforation, continue to occur suddenly.^[5,6]^

Peptic ulcer perforation (PUP) constitutes 5% of all acute abdominal patients as PUP complications.^[7]^ If the diagnosis is delayed, serious complications such as localized or generalized peritonitis, sepsis, and even death may occur.^[7,8]^ There is not any factor that can easily recognize the patients with high mortality risk. Old age, presence of comorbidity, and delay in surgery increase the risk of mortality.^[8]^ The mortality rate ranges between 10% and 40%.^[5,9]^ Therefore, surgery is still considered the most appropriate treatment for PUP.^[7,8]^ However, despite the surgical treatment of PUP, high morbidity and mortality rates prompted researchers to search for new laboratory markers for predicting prognosis.

There is no standard criterion for PUP diagnosis. Patient history is the most important factor in determining diagnosis. The diagnosis becomes difficult in patients without a history of PUD. PUP patients range from those who are completely asymptomatic to those who develop serious complications. However, peritonitis findings such as sudden onset of severe abdominal pain, vomiting, shock, and defensive-rebound may help to diagnose perforation.^[10]^ For PUP, free air below the diaphragm may be diagnostic in the patient who has a history of PUD. Surgery is the best choice for his treatment. The delay in diagnosis and surgery is a cause of high morbidity and mortality.^[10,11]^

Biomarkers, C-reactive protein (CRP), procalcitonin, mean platelet volume, platelet/lymphocyte ratio (PLR), and neutrophil/lymphocyte ratio (NLR) have been used as inflammatory markers to predict prognosis in inflammatory diseases.^[7,8]^ NLR and PLR have been proposed as markers of systemic inflammation in clinical practice. Malignancy, coronary artery disease, acute appendicitis, acute cholecystitis, acute pancreatitis, and PUP are increasingly used in predicting prognosis.^[12–14]^ Serum albumin is closely related to the degree of malnutrition and is an indicator of regular nutritional status. Loss of albumin may be seen in the inflammatory response. Also, abnormal serum albumin has been reported to be closely related to the progression of many diseases.

Studies use NLR and PLR values to determine postoperative patient prognosis and mortality risk in patients treated with PUP surgery.^[15]^ Because the amount of platelets and albumin in the blood is measured before surgery, it does not require additional costs. This study aims to investigate the relationship between preoperative platelet/albumin ratio (PAR) and postoperative mortality in patients operated for PUP.

## 2. Materials and Methods

### 2.1. Study design

This retrospective study included 171 patients operated for PUP at the Dicle University Medical Faculty General Surgery Clinic between June 2013 and December 2019. The study was approved by the Clinical Research Ethics Committee of Dicle University in accordance with the Declaration of Helsinki.

The inclusion criteria:

1. Laboratory parameters including platelet and albumin values measured before surgery at the time of diagnosis.
2. Complete clinical and pathological features and follow-up data.

Exclusion criteria:

1. Cancer treatment history.
2. Acute or chronic inflammatory disease.
3. Malignancy as a result of pathology.
4. Hemolysis blood sample.

### 2.2. Data collection

The main clinical characteristics such as age, gender, location of the perforation, American Society of Anesthesiologists score, serum levels of platelet, albumin were extracted from retrospective medical records. Routine laboratory measurements, including counts of white blood cells, neutrophils, and lymphocytes counts were performed before treatment. Albumin value was correlated with aspartate aminotransferase, alanine aminotransferase, and potassium values and was excluded in the presence of possible hemolysis. The patients were divided into 2 groups: PUP-S (patients who survived after surgical treatment) and PUP-M (patients who died after surgical treatment). The clinical and laboratory features were categorized in the comparisons of the groups.

### 2.3. Statistical analysis

The data were analyzed using SPSS Statistics version 22. Data distribution was evaluated using the Kolmogorov–Smirnov test. Continuous variables were expressed as means with standard deviations (SD) or medians with ranges. Categorical variables were expressed as percentage frequency. The Mann–Whitney *U* test was used for analyzing the nonparametric data and the independent samples *t* test was applied for the parametric data. The Spearman correlation analyses were used for determining correlations between the parameters of the patients. The *P* value of <.05 was considered statistically significant.

## 3. Results

The average age of the patients was 46.3 ± 20.5 years. Thirty-three patients (19.3%) were female and 138 (80.7%) were male. The average length of hospital stay is 9.71 ± 7.10 days. The surgical primary repair and omentoplasty were performed on all patients. Open surgery was performed in 152 patients, while laparoscopic surgery was performed in 19 patients. Mortality was not observed in any patient who had undergone laparoscopic surgery. The patients were divided into 2 groups as PUP-S (n = 144) and PUP-M (n = 27).

Hospitalization time, preoperative white blood cell, platelets, mean platelet volume, neutrophil, and NLR values were not statistically different between the groups (Table 1). The mean age was significantly higher in the PUP-M group (71.2 ± 15.7 years) than in the PUP-S group (41.7 ± 17.8 years; *P* ≤ .001). Preoperative lymphocyte count was 1.24 ± 1.38 in the PUP-M group and 2.10 ± 4.94 in the PUP-S group. The difference was statistically significant (*P* = .006). The preoperative PLR was higher in the PUP-M group (463.6 ± 394.5) than in the PUP-S group (248.6 ± 185.6, *P* = .02). Preoperative lactic dehydrogenase (LDH)/albumin ratio was higher in the PUP-M group (210.1 ± 154.3) than in the PUP-S group (73.0 ± 43.6, *P* ≤ .001). Preoperative PAR was higher in the PUP-M group (149.9 ± 83.9) than in the PUP-S group (79.8 ± 37.6, *P* ≤ .001). A statistically significant difference was found between groups according to the age, albumin, and LDH values (Table 1).

**Table 1:** Results of the statistical comparisons of the variables of the groups.

**Characteristic**	**Total patient (n = 171), mean ± SD**	**PUP-S (n = 144), mean ± SD**	**PUP-M (n = 27), mean ± SD**	***P* value** ^*^
Age (yr)	46.3 ± 20.5	41.7 ± 17.8	71.2 ± 15.7	≤.001
Length of stay in hospital (d)	9.71 ± 7.10	8.97 ± 5.27	13.6 ± 12.5	.196
Albumin (g/dL)	3.31 ± 0.91	3.56 ± 0.76	2.00 ± 0.42	≤.001
LDH (U/L)	266.6 ± 143.5	240.8 ± 87.8	404.2 ± 262.4	≤.001
CRP (mg/dL)	6.64 ± 8.70	5.27 ± 7.53	13.9 ± 10.8	≤.001
WBC (×10^9^/L)	13.8 ± 5.92	14.2 ± 5.76	12.1 ± 6.59	.093
Hemoglobin (g/dL)	13.9 ± 2.66	14.5 ± 2.37	11.2 ± 2.52	≤.001
Hematocrit (%)	42.3 ± 7.77	43.6 ± 6.85	35.3 ± 8.67	≤.001
Platelet (×10^3^/µL)	271.4 ± 109.5	267.4 ± 97.7	292.9 ± 159.4	.794
MPV (fL)	9.86 ± 20.8	10.2 ± 22.7	8.06 ± 1.64	.683
Neutrophil (×10^3^/µL)	11.4 ± 5.72	11.6 ± 5.64	10.5 ± 6.13	.356
Lymphocyte (×10^3^/µL)	1.96 ± 4.58	2.10 ± 4.94	1.24 ± 1.38	.006
Neutrophil/lymphocyte	11.8 ± 11.1	11.4 ± 11.0	14.4 ± 11.3	.091
LDH/albumin	94.7 ± 88.0	73,0 ± 43.6	210.1 ± 154.3	≤.001
Platelet/albumin ratio	90.9 ± 54.0	79.8 ± 37.6	149.9 ± 83.9	≤.001
Platelet/lymphocyte ratio	282.5 ± 24.28	248.6 ± 185.6	463.6 ± 394.5	.002
Clavien–Dindo classification				
<III	143 (83.6%)	133 (92.3%)	10 (37%)	≤ 0.001
≥III	28 (16.4%)	11 (7.7%)	17 (63%)	

According to the results of correlation analysis positive correlation was detected between length of stay in hospital and age (*r* = 0.306, *P* ≤ .001), platelets (*r* = 0.154, *P* = .044), LDH/albumin ratio (*r* = 0.227, *P* ≤ .001), and PAR (*r* = 0.334, *P* ≤ .001). A negative correlation was found between albumin and length of stay in hospital (*r* = –329, *P* ≤ .001; Table 2).

**Table 2:** Correlation analysis (spearman test).

	**Length of stay in hospital**	
	***r***	***P* value**
Age (yr)	0.306	≤.001
Albumin (g/dL)	–0.329	≤.001
LDH (U/L)	0.115	.135
CRP (mg/dL)	0.188	.014
WBC (×10^9^/L)	–0.113	.140
Hemoglobin (g/dL)	–0.114	.139
Hematocrit (%)	–0.101	.189
Platelet (×10^3^/µL)	0.154	.044
MPV (fL)	–0.086	.264
Neutrophil (×10^3^/µL)	–0.088	.250
Lymphocyte (×10^3^/µL)	–0.078	.313
Neutrophil/lymphocyte ratio	–0.025	.750
LDH/albumin ratio	0.279	≤.001
Platelet/albumin ratio	0.341	≤.001
Platelet/lymphocyte ratio	0.122	.113

Mortality developed in 27 patients (15.7%) in the postoperative period. The most common cause of death was sepsis in 19 patients. Death occurred due to pulmonary complications in 4 patients, acute renal failure in 2 patients, cerebral hemorrhage in 1 patient, and myocardial infarction in 1 patient.

## 4. Discussion

PUD is the most common gastrointestinal disease. PUP, which develops as a PUD complication, is most common among the individuals aged 40 to 50 years. PUP forms the acute abdomen and may cause peritonitis, sepsis, and death.^[8]^ Although it is more common in men, the male/female ratio varies. Mortality and morbidity increase with age.^[16,17]^

In the retrospective study of Taş et al^[18]^ the average patient age was 51.7 years, and it was found it to be 39.5 years in the study of Ugochukwu et al.^[10]^ In our study, the average patient age was 46.3, consistent with the other studies.

Since PUP operations are associated with high morbidity and mortality rates, it is very important to identify diagnostic markers and risk factors affecting perforation. These risk factors can be listed as male gender, over 65 years of age, comorbidity, PUD history, alcohol consumption, use of nonsteroidal anti-inflammatory drugs, long hospital admission time, presence of shock findings, and BUN/creatinine ratio >12.^[7,18,19]^ Testini et al^[20]^ reported that the risk of postoperative mortality was higher in PUP patients aged >65 years compared to younger patients. In our study, compatible with the literature, the mean age of patients who developed mortality was 71.2.

PUP is usually diagnosed clinically. The clinical diagnostic indicators of PUP are sudden inceptive abdominal pain and tenderness. Although perforation on the anterior surface of the stomach usually results in intense abdominal pain, perforation on the posterior surface often manifests as back pain.^[10]^ Radiological diagnosis is made on the x-ray by subdiaphragmatic free air.

Subdiafragmatic free air was reported in the abdomen x-ray images of patients with PUP with a rate of 47.2% to 80%.^[17]^ However, several case series showed that x-ray may be negative for free air, especially in elderly patients.^[21]^ We diagnosed the patients with abdominal pain by chest X-ray.

High morbidity and mortality rates due to the delay in diagnosis and surgical treatment of PUP encouraged researchers to develop new laboratory tests and imaging methods. In 2013, Jafarzadeh and colleagues^[22]^ published a correlation between Helicobacter *pylori*, white blood cell count and NLR. In another study, it was found that *H pylori* eradication reduced blood neutrophil and monocyte counts.^[23]^ In our study, we found high PAR and CRP values in PUP-M patients.

The hypothesis of the study is the fact that high platelet and low albumin levels are associated with poor prognosis in many patients has been created with the question of how PAR will affect prognosis. Serum albumin level is an important marker of malnutrition. Recent studies have shown that albumin is a systemic factor that reflects both nutritional and chronic inflammatory conditions. Therefore, low albumin value is also a poor prognostic factor in PUP patients. However, albumin is still a controversial marker for prognosis.^[24–26]^ Also, hypoalbuminemia significantly affects hospital stay and complication rates.^[27–29]^ Platelets play a critical role in hemostasis and are also important in the development of pathological processes such as atherosclerosis and arterial thrombosis. High (thrombocytosis) platelet counts are common findings in a variety of diseases, including liver diseases, infections, autoimmune disorders, and malignancies. The morbidity and mortality due to abnormal platelet counts are generally thought to be due to such underlying diseases. We found that, when it was used to describe the PUP, mortality was also high in patients with high PAR. Delaying the diagnosis and treatment of patients with PUP is life-threatening.

Therefore, differential diagnosis of PUP should be rapid. Undoubtedly, the effect of PAR on prognosis should be supported by studies with prospective, large patient groups. However, easy and cost-formed PAR may increase the use of markers in these patients.

### 4.1. Limitations of the study

Our study has some limitations.

1. There was a big difference between the number of patients in the groups.
2. It was planned retrospectively.
3. There was no comparison between the hemoglobin values and thrombocyte values of the patients.
4. There was an imbalance between the groups. Most of the patients who died were elderly and they had comorbidities.
5. It was aimed to define the predictive and prognostic parameters obtained from routine biochemical laboratory tests in patients operated on for PUP diagnosis. Therefore, the radiological images of the patients were not evaluated.

## 5. Conclusion

High PAR value is a negative risk factor for mortality and length of hospital stay in patients operated for PUP. Also, it has been determined that mortality rates can be high in patients with high LDH/albumin ratio and PLR values and low lymphocyte and albumin count. Our results should be supported by prospective, large patient groups and long follow-up studies. However, in the future, PAR can be used as a parameter for clinical use in predicting mortality and morbidity of PUP.

## Footnotes

* Mann–Whitney *U* test, *P* < .05 statistically significant.
